# Evaluation of risk factors associated with drug-resistant tuberculosis in Yemen: data from centres with high drug resistance

**DOI:** 10.1186/s12879-019-4069-1

**Published:** 2019-05-24

**Authors:** Ammar Ali Saleh Jaber, Baharudin Ibrahim

**Affiliations:** 1 Department of Clinical Pharmacy, School of Pharmaceutical Sciences, Universiti Sains Malaysia, Penang, Malaysia; 2 Department of Clinical Pharmacy, Faculty of Pharmacy, Taiz University, Taiz, Yemen

**Keywords:** Drug resistance, Khat, Tuberculosis, Yemen

## Abstract

**Background:**

The World Health Organization (WHO) has reported that Yemen has a high burden of drug resistance and a worrying shortage of implemented diagnostic methods and drug treatment regimens. Therefore, in this study, we evaluated the risk factors associated with multidrug-resistant tuberculosis (MDR-TB) and explored the poor TB management in Yemen.

**Methods:**

Between January 2014 and December 2016, we enrolled 135 patients with MDR-TB from drug resistance programmes at four major TB centres in Yemen for this prospective study. After exclusion of 20 patients, treatment outcomes were reported for 115 patients who attended a series of follow-ups.

**Results:**

A total of 115 patients with MDR-TB were analysed from the four main TB centres in Yemen. Most patients (35.2%) were from the Aden TB centre. A success rate of 77.4% was reported for TB treatment. Of the 115 patients, 69.6% were resistant to two drugs, 18.3% were resistant to three drugs, and 12.2% were resistant to four drugs. During the intensive phase of treatment, 19 patients (16.5%) reported one or more adverse events. A multivariate logistic regression analysis revealed that a baseline body weight of ≤40 kg [*p* = 0.016; adjusted odds ratio (AOR) = 25.09], comorbidity (*p* = 0.049; AOR = 4.73), baseline lung cavities (*p* = 0.004; AOR = 15.32), and positive culture at the end of the intensive phase (*p* = 0.009; AOR = 8.83) were associated with the unsuccessful treatment outcomes in drug-resistant TB patients.

**Conclusions:**

The success rate achieved after treatment was below the levels established by the WHO End TB Strategy (90%) and the United Nations Sustainable Development Goals (80%). Identification of risk factors associated with MDR-TB in Yemen is essential because it allows health workers to identify high-risk patients, especially in the absence of a second-line treatment or a laboratory diagnostic method. The Yemen National Tuberculosis Control Program should formulate new strategies for early detection of MDR-TB and invest in new programmes for MDR-TB management.

## Background

Since the introduction of the first drug treatment for tuberculosis (TB) in 1943, drug resistance has increased. Following the widespread use of rifampicin, which began in the 1970s, the increased incidence of multidrug-resistant TB (MDR-TB), which refers to the resistance to isoniazid and rifampicin [1], has led to the use of second-line drugs. The improper use of these drugs has fuelled the emergence and subsequent transmission of extensively drug-resistant TB, which is resistant to at least one fluoroquinolone drug and to an injectable agent, in addition to isoniazid and rifampicin [2].

TB is one of the leading causes of death worldwide [3]. In Yemen, a country in the eastern Mediterranean region, with a high TB burden, the disease is considered a major public health problem, ranking fourth on the list of public health priorities. In 2017, the World Health Organization (WHO) reported that Yemen had 13,000 new TB cases, with an incidence rate of 47 cases per 100,000 individuals [4]. According to a 2011 TB study in Yemen, the percentages of newly and previously treated MDR-TB cases were 2.4 and 19%, respectively [5]. Among new cases, the success rate of treatment was 85%, and among previous patients, it was 75%; a low success rate (72%) was achieved with rifampicin and in patients with MDR-TB [5]. Only one survey was conducted in Yemen to determine the prevalence of MDR-TB in 2004. The study reported that 14 of 511 (2.7%) newly treated and seven of 54 (12.9%) previously treated patients had MDR-TB [6].

Factors such as limited availability of free and second-line drugs in developing countries such as Yemen, a prolonged duration of treatment, high prices, treatment-associated toxicity, the lack of specialists and laboratory facilities, and the practice of selling second-line TB drugs in private sectors hinder the effective characterisation of treatment outcomes of drug resistance therapy [7–11]. Because of the civil war in Yemen, conditions have recently worsened, and information regarding MDR-TB is scarce. Moreover, treatment outcomes and disease management have not been explored in this country. In this study, we aimed to evaluate the risk factors associated with MDR-TB and to explore the poor TB management in Yemen. The current study is, therefore, the first to report treatment outcomes of MDR-TB in Yemen and its associated risk factors.

## Methods

### Study population

**Fig. 1:**
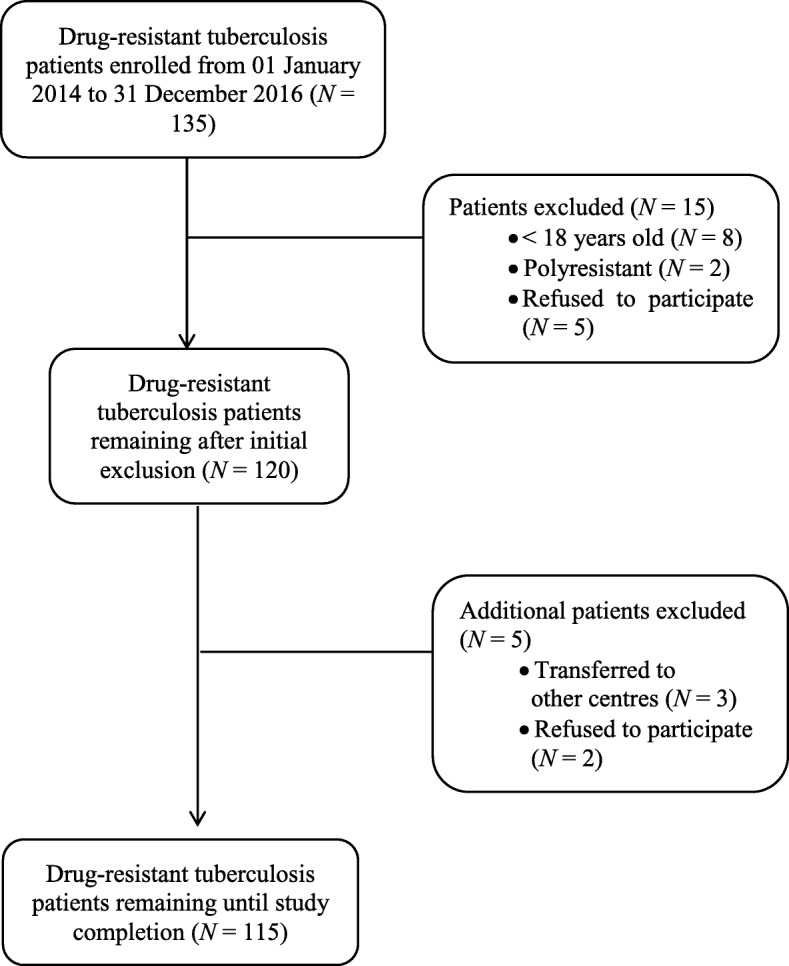
Flowchart of the number of MDR-TB patients enrolled in the study

This prospective observational study was conducted in four major TB centres, namely Al-Hudaydah, Taiz, Aden, and Sana’a, which are among the largest in Yemen. Only TB culture is performed in these centres for diagnosis. A total of 135 patients with MDR-TB were enrolled between 01 January 2014 and 31 December 2016 (Fig. 1). Most of the MDR-TB patients (115) had follow-up visits until treatment outcomes were reported. Patients who were younger than 18 years and polyresistant (resistant to more than one first-line anti-TB drug, other than isoniazid and rifampicin), as well as those who refused to participate, were excluded from the study.

The study was approved by the Yemen Ministry of Public Health and National Tuberculosis Control Program (NTCP).

### Bacteriology and drug susceptibility testing

The drug-resistant TB suspects referred to the study site were initially evaluated using two sputum samples. After positive direct sputum smear microscopy for acid-fast bacilli (AFB) using Ziehl–Neelsen staining and rapid testing for *Mycobacterium tuberculosis* and rifampicin resistance using the Xpert MTB/RIF assay (Cepheid, Sunnyvale, CA, USA), sputum samples were sent to the Sana’a TB centre laboratory for sputum culture and drug susceptibility testing (DST). DST is only available at the main TB centre in Yemen, which is located in Sana’a. DST was performed at baseline and repeated whenever deemed necessary; AFB sputum smear and culture were performed every month.

### Treatment of multidrug-resistant TB in Yemen

Based on the guideline for MDR management in Yemen, MDR-TB treatment is under category 3 [1]. The bacteriological examination of MDR-TB includes sputum microscopy and culture. To confirm TB, we performed sputum microscopy at the beginning of treatment based on the infectiousness. At least one sputum sample for smear and culture was collected at the start of each treatment. To consider MDR-TB under category 3, at least one pretreatment culture or smear must be positive. Collection of samples for culturing or smearing was performed less than 30 days before or 7 days after the initiation of the categorical treatment. AFB sputum smears and cultures were used to monitor patients on a monthly basis throughout the therapy period.

### Data collection

The sociodemographics of the patients, laboratory test results, and clinical data were obtained using a standardised data collection form. Information regarding adverse effect was obtained from drug-resistant patients, patients’ treatment cards, and TB centre records. Sputum conversion results were obtained from the laboratories of the TB centres. Each patient had a unique code for follow-up laboratory tests.

### Treatment outcomes

Final treatment outcomes were classified into two categories (successful or unsuccessful). Successful treatment outcomes were further reported as either ‘cured’ or ‘completed’. Unsuccessful treatment outcomes were reported when patients experienced treatment failure, died during treatment from any cause, were lost to follow-up, or were not evaluated.

### Statistical analysis

Data were analysed using the PASW software version 24 (IBM Corp, Armonk, NY, USA). All categorical data were reported as a percentage and number. Univariate logistic regression was used to identify the risk factors associated with unfavourable treatment outcomes. Significant values were added to multivariate logistic regression to determine the final risk factors. Significance was defined as a *p*-value of less than 0.05. Odds ratio (OR), adjusted odds ratio (AOR), and confidence interval were also calculated.

## Results

### Treatment regimens for MDR-TB patients in Yemen

Treatment for MDR-TB in Yemen lasts for at least 20 months after culture conversion, but an extension to 24 months may be indicated for patients with extensive pulmonary damage. Table 1 lists the treatment regimens used in Yemen.

**Table 1:** Treatment regimens for MDR-TB patients in Yemen

Resistance to	Treatment regimen
RHSE	Regimen 1 8 months of amikacin + levofloxacin + ethionamide + cycloserine + *para*-aminosalicylic acid, followed by 16 months of levofloxacin + ethionamide + cycloserine + *para*-aminosalicylic acid
RHS	Regimen 2 8 months of amikacin + levofloxacin + ethionamide + cycloserine + pyrazinamide, followed by 16 months of levofloxacin + ethionamide + cycloserine + pyrazinamide
RH	Regimen 3 8 months of amikacin + levofloxacin + ethionamide + cycloserine + ethambutol, followed by 16 months of levofloxacin + ethionamide + cycloserine + ethambutol

Abbreviations: *R,* rifampicin; *H,* isoniazid; *S,* streptomycin; *E,* ethambutol

### Patient characteristics

Of the 135 MDR-TB patients initially registered at the four main TB centres in Yemen, only 115 fulfilled the inclusion criteria and were thus included in the final analysis (Fig. 1). Sixty-five (56.5%) of the patients were males, and 35.7% of the patients were from the Aden TB centre. Additionally, 60.9% (70) of the patients were of reproductive age (≤ 45 years), and 73% (84) were married. Although the study was conducted in an urban area, approximately 40% (46) of patients travelled from rural areas. Most patients (104, or 90.4%) chewed khat, and 73.9% (85) were smokers. Approximately 73% (84) of the patients lived under the poverty line as their monthly income was less than 50% of the threshold. Illiteracy was found to be high (67%), and 5.2% (6) of the patients had previously received TB treatment, while 94.8% (109) were newly diagnosed. During the intensive phase of treatment, 26.1% (30) of patients experienced adverse effects, and 12.2% (14) changed their treatment regimens because of adverse effects (Table 2).

**Table 2:** Characteristics of the MDR-TB patients (*N* = 115)

Characteristics	Patients, *N* (%)
Centre	
Al-Hudaydah	37 (32.2)
Taiz	17 (14.8)
Sana’a	20 (17.4)
Aden	41 (35.7)
Sex	
Male	65 (56.5)
Female	50 (43.5)
Age (years)	
≤ 45	70 (60.9)
> 45	45 (39.1)
Marital status	
Married	84 (73)
Unmarried	31 (27)
Area	
Rural	46 (40)
Urban	69 (60)
Household size	
≤ 7	40 (34.8)
> 7	75 (65.2)
Smoking status	
Non-smoker	30 (26.1)
Smoker	85 (73.9)
Chewing khat^a^	
Yes	104 (90.4)
No	11 (9.6)
Literacy status	
Illiterate	77 (67)
Literate	38 (33)
Employment status	
Employed	31 (27)
Unemployed	84 (73)
Monthly income (rials^b^)	
≤ 10,000	31 (27)
> 10,000	84 (73)
Baseline weight (kg)	
≤ 40	36 (31.3)
> 40	79 (68.7)
BCG vaccination^c^	
No	21 (18.3)
Yes	94 (81.7)
History of TB treatment	
Previously treated	6 (5.2)
Newly treated	109 (94.8)
History of streptomycin use	
No	92 (80)
Yes	23 (20)
History of failed TB treatment	
No	63 (54.8)
Yes	52 (45.2)
Family history of TB	
Drug-susceptible TB	99 (86.1)
MDR-TB	16 (13.9)
Adverse events at the end of the intensive phase	
No	85 (73.9)
Yes	30 (26.1)
Regimen changes based on adverse events	
No	101 (87.8)
Yes	14 (12.2)

^a^Khat is a shrub that grows in parts of East Africa and Yemen

^b^Rial, one dollar is equivalent to approximately 215 rials

^c^Bacillus Calmette–Guerin (BCG) is a vaccine for TB

### Treatment outcomes

At treatment completion, the success rate was 77.4% (29.6% cured and 47.8% completed), while unsuccessful treatment outcomes were indicated for 22.6% of patients. Out of the total (115) patients, 14 (12.2%) died, six (5.2%) were lost to follow-up, four (3.5%) experienced treatment failure, and data from two patients (1.7%) were not evaluated (Table 3).

**Table 3:** Treatment outcomes in the MDR-TB patients based on the WHO/International Union Against Tuberculosis and Lung Disease criteria

Treatment outcome	Patients, *N* (%)
Successful	89 (77.4)
Cured	34 (29.6)
Completed	55 (47.8)
Unsuccessful	26 (22.6)
Lost to follow-up	6 (5.2)
Not evaluated	2 (1.7)
Died	14 (12.2)
Failed	4 (3.5)
Total	115 (100)

### Clinical outcome-related patient characteristics

Table 4 shows the clinical characteristics of patients with MDR-TB. Approximately 59.1% had a high sputum grade at the beginning of treatment, and 40.9% displayed baseline lung cavities. Moreover, 41.1% reported sputum conversion at the end of the second month of treatment, and 59.1% of the patients had haemoglobin levels below normal. Thirty patients (26.1%) also had comorbidities, including 12 patients with diabetes, eight with hypertension, five with HIV infection, and two with chronic obstructive pulmonary disease.

**Table 4:** Clinical characteristics and drug resistance patterns of the patients (*N* = 115)

Variable	Patients, *N* (%)
Sputum grading	
Low	47 (40.9)
High	68 (59.1)
Baseline lung cavity	
No	68 (59.1)
Yes	47 (40.9)
Positive culture at the end of the second month	
No	67 (58.3)
Yes	48 (41.7)
Creatinine level	
Normal^α^	105 (91.3)
Above normal	10 (8.7)
Hb level	
Normal^α^	47 (40.9)
Below normal	68 (59.1)
WBC count	
Normal^α^	78 (67.8)
Above normal	37 (3.2)
Bilirubin level	
Normal^α^	106 (92.2)
Above normal	9 (7.8)
Comorbidity	
No	85 (73.9)
Yes	30 (26.1)
Type of comorbidity	
Diabetes mellitus	12 (40)
Hepatitis	3 (10)
Hypertension	8 (26.7)
Chronic obstructive pulmonary disease	2 (6.7)
HIV	5 (16.6)
Resistance to TB drugs	
HR	80 (69.6)
HRE	11 (9.6)
HRS	10 (8.7)
HRES	14 (12.1)

^**α**^Normal ranges: Hb > 13 g/dL (males) and > 11.5 g/dL (females); WBCs > 11,000/mm^3^; creatinine < 1.1 mg/dL (males) and < 0.9 mg/dL(females); bilirubin ≤1 mg/dL

Abbreviations: *Hb*, haemoglobin; *WBC*, white blood cell; *H*, isoniazid; *R*, rifampicin; *E*, ethambutol; *S*, streptomycin

### Resistance patterns

Most of the patients (69.6%) were resistant to two drugs, while 21 (18.3%) patients were resistant to three drugs and 14 (12.2%) patients were resistant to four drugs (Table 4).

### Adverse events during the intensive phase

Nineteen patients (26.1%) experienced one or more adverse events during the intensive phase of treatment, with gastrointestinal effects (13.3%) being the most dominant. Approximately 6.7% of patients reported arthralgia, hearing disturbance, hepatic toxicity, and haematological reactions, and 3.3% reported psychiatric disorders, renal toxicity, peripheral toxicity, and dermatological reactions (Table 5).

**Table 5:** Adverse events associated with MDR-TB treatment during the intensive phase (*N* = 19)

Adverse events	Patients^a^, *N* (%)
Gastrointestinal effects	4 (13.3)
Arthralgia	2 (6.7)
Psychiatric disorders	1 (3.3)
Hearing disturbance	2 (6.7)
Renal toxicity	1 (3.3)
Peripheral neuropathy	1 (3.3)
Hepatic toxicity	2 (6.7)
Dermatological reactions	1 (3.3)
Haematological reactions	2 (6.7)
Others	3 (10)

^**a**^Drug-resistant patients with more than one adverse reaction

### Predictors of unsuccessful treatment outcomes

Univariate analysis revealed that a baseline body weight of ≤40 kg (*p* = 0.031; OR = 4.1), comorbidity (*p* = 0.044; OR = 18.8), baseline lung cavities (*p* = 0.036; OR = 17.9), and positive culture at the end of the intensive phase of treatment (*p* = 0.016; OR = 14) were associated with unsuccessful treatment outcomes in drug-resistant patients (Table 6). When these significant variables from the univariate analysis were included in the multivariate analysis, baseline body weight of ≤40 kg (*p* = 0.016; AOR = 25.09), comorbidity (*p* = 0.049; AOR = 4.73), baseline lung cavities (*p* = 0.004; AOR = 15.32), and positive culture at the end of the intensive phase of treatment (*p* = 0.009; AOR = 8.83) were also found to be associated with unsuccessful treatment outcomes in the MDR-TB patients (Table 7).

**Table 6:** Predictors of unsuccessful treatment outcomes, as determined by simple logistic regression

Variable	Treatment outcome		β	SE	*p*-Value^a^	OR (95% CI)
	Unsuccessful (*N* = 26)	Successful (*N* = 89)				
Age (years)						
≤ 45	8	62				Ref
> 45	18	27	0.722	1.52	0.636	0.486 (0.24–9.7)
Baseline weight (kg)						
> 40	13	23				Ref
≤ 40	13	66	3.83	1.77	0.031	4.1 (1.43–14.8)
Comorbidity						
No	7	78				Ref
Yes	19	11	2.939	1.461	0.044	18.8 (1.01–33.7)
Baseline lung cavities						
No	2	66				Ref
Yes	23	24	2.889	1.377	0.036	17.9 (1.20–26.7)
Creatinine level						
Normal	17	88				Ref
Above normal	1	9	−0.347	1.11	0.75	0.71 (0.081–6.2)
History of streptomycin use						
No	18	74				Ref
Yes	8	15	1.99	1.210	0.100	7.3 (0.68–78)
Positive culture, end of I.P.						
No	64	3				Ref
Yes	25	23	2.667	1.112	0.016	14 (1.62–127)
History of failed TB treatment						
No	5	58				Ref
Yes	21	31	1.174	0.975	0.229	3.23 (0.47–21.8)
Smoking status						
No	1	29				Ref
Yes	25	60	1.757	1.55	0.259	3.2 (0.47–21.87)
Chewing khat^b^						
No	2	9				Ref
Yes	24	80	−0.806	2.043	0.693	0.4 (0.008–24.5)
Resistance to FLDs						
≤ 2	17	60				Ref
> 2	9	29	−1.11	0.898	0.215	0.3 (0.05–1.911)

^a^*p*-Values ≤0.05 are shown in bold

^b^Khat is a shrub that grows in parts of East Africa and Yemen

Abbreviations: *SE*, standard error of the mean; *OR*, odds ratio; *CI*, confidence interval; *Ref*, reference group; *I.P.*, intensive phase; *FLD*, first-line drug

**Table 7:** Predictors of unsuccessful treatment outcomes, as determined by multiple logistic regression

Variable	β	SE	*p*-Value	AOR	95% CI
Comorbidity	1.556	0.790	0.049	4.73	1.00–22.30
Baseline lung cavities	2.729	0.935	0.004	15.32	2.45–95.8
Baseline weight ≤ 40 kg	3.223	1.344	0.016	25.09	1.80–34.9
Positive culture, end of I.P.	2.178	0.830	0.009	8.83	1.73–44.9

Abbreviations: *SE*, standard error of the mean; *AOR*, adjusted odds ratio; *CI*, confidence interval; *I.P.*, intensive phase

## Discussion

At the end of the treatment period, only 89 (77.4%) of the 115 patients achieved success, and thus, this study did not reach the success rate established by the United Nations Sustainable Development Goals (80%) and the WHO End TB Strategy (90%). The low success rate in the present study was due to a high mortality rate (12.2%) and loss to follow-up (5.2%). The high mortality rate in our study might have been due to various factors, such as the late detection of MDR-TB and the disease, late initiation of the treatment course, a low educational level, a high number of previous TB episodes, the presence of comorbid conditions, especially diabetes mellitus, insufficient treatment, and unavailability of modern laboratory equipment for detecting resistance to other second-line TB drugs [12]. Nine countries (Afghanistan, Burkina Faso, Chad, Congo, Papua New Guinea, Sierra Leone, Somalia, South Sudan, and Yemen), with more than 5000 TB cases registered in 2015, reported that they did not have the capability to perform phenotypic DST [13], and similar studies have reported high mortality rates in the Dominican Republic and Russia (11.3 and 10.9%, respectively) [14, 15]. The NTCP should therefore formulate new strategies for early detection of MDR-TB and invest in new treatments for MDR-TB management. Based on the literature, approximately 26% of patients are lost to follow-up because of drug toxicity. Other factors include poor knowledge/understanding of MDR-TB, high levels of poverty, a low economic status, low family support, and unsatisfactory health services available at main centres [16–18]. To improve the TB healthcare system in Yemen, the following measures should be adopted: the provision of adequate training for health care providers and counselling for patients, retrieval of the exact address for the parents of patients, establishment of a proper drug supply, provision of free food and transportation for patients and caregivers, dissemination of health educational materials by health workers, and initiation of home visits to eliminate the lost to follow-up treatment outcome.

In the present study, a baseline weight of ≤40 kg was associated with unsuccessful treatment outcomes, which is consistent with the data of other studies [19–21]. Body mass index is considered an accurate measure for determining whether a patient is underweight. However, since patient height was not measured, this parameter could not be determined. Most of the population in Yemen is below the poverty line [22], earning an income below 50 US dollars per month [23]. Therefore, patients have a low body weight, which can be associated with low immunity, and this can lead to TB displaying longer than usual manifestations in these patients [20]. Initiating food programmes for MDR-TB patients may increase their weight, consequently improving their immunity and the success rate of treatment.

This study showed baseline cavitation to be a risk factor associated with unsuccessful treatment outcomes, which may be due to a low efficacy of anti-TB drugs. Lung cavitation at baseline is a poor predictor of treatment outcome [14, 18, 19, 24]. In the present study, a regimen modification due to a severe adverse event was reported for 12.2% of patients, which was lower than the percentages (47 and 45% of patients) reported in Iran and Latvia, respectively [25, 26]. As reported, MDR-TB regimen modifications are predictors of unsuccessful treatment outcomes [26]. Low bioavailability of a second-line drug, which is considered less potent than first-line drugs, may be the reason for the association between regimen modification and unfavourable treatment outcome [26]. This important finding suggests that early detection and management of adverse effects may improve treatment outcomes [25].

In this study, comorbidities, predominantly diabetes mellitus, were also found to be a risk factor associated with unsuccessful treatment outcomes. Similar studies have also reported diabetes mellitus as a risk factor for these patients [12, 27]. Diabetes mellitus lowers one’s immunity, thereby increasing the strength of TB infection and reducing the effectiveness of a TB drug. Moreover, the complex medication schedule for diabetes and TB may lead to low adherence of patients to the schedule [12]. Early detection and management of comorbid conditions, especially diabetes mellitus, may therefore improve the outcomes of these patients.

Negative culture at the end of the second month of MDR-TB treatment indicates the high effectiveness of a TB drug and is an early sign of successful treatment [28]. Various studies have reported a high success rate for those who achieved negative culture at the end of the second month of treatment [18, 24]. Our study reported positive sputum cultures after the second month of treatment, indicating an unsuccessful treatment outcome, similar to the data of some other studies [14, 18, 29, 30]. Based on this result, obtaining a positive culture at the end of the second month of treatment may help health workers identify TB patients with potentially unsuccessful outcomes. This variable can also act as a reliable indicator of treatment efficacy.

This study had some limitations. The study was conducted in four main TB centres in Yemen, with its major limitation being a small number of participating patients. Given, however, that this study included patients from an extensive geographical area, the findings may reflect the treatment outcomes of MDR-TB and the associated risk factors across Yemen. Nonetheless, a multi-centre study with a large sample size and drug-susceptible TB controls is needed to corroborate the findings.

## Conclusions

By knowing the risk factors associated with unfavourable treatment outcomes, health workers can easily identify patients with a high risk of death or those who are likely to be lost to follow-up during treatment. The clinical outcomes and clinical management of MDR-TB can therefore be improved by considering these factors. Initiating new food programmes may increase the body weight and immunity of patients, while establishing new strategies for identifying and managing diabetes may increase their immunity.

The NTCP should formulate new strategies for early detection of MDR-TB and invest in new programmes for MDR-TB management. Early detection of adverse effects of TB drugs allows drug regimen modification during treatment, which in turn improves patient outcomes.

Despite their extensive efforts, health workers and the NTCP in Yemen have had little success in improving the treatment outcomes of patients with MDR-TB. We believe that the success rate of TB treatment in Yemen may decrease even further because of the instability caused by the current war. Since treatment was reported to be unavailable, non-government organisations must increase their efforts to fulfil this void for the prevention of future disasters.

## Abbreviations

AFB: Acid-fast bacilli
AOR: Adjusted odds ratio
DST: Drug susceptibility testing
MDR-TB: Multidrug-resistant tuberculosis
NTCP: National Tuberculosis Control Program
OR: Odds ratio
TB: Tuberculosis
WHO: World Health Organization
